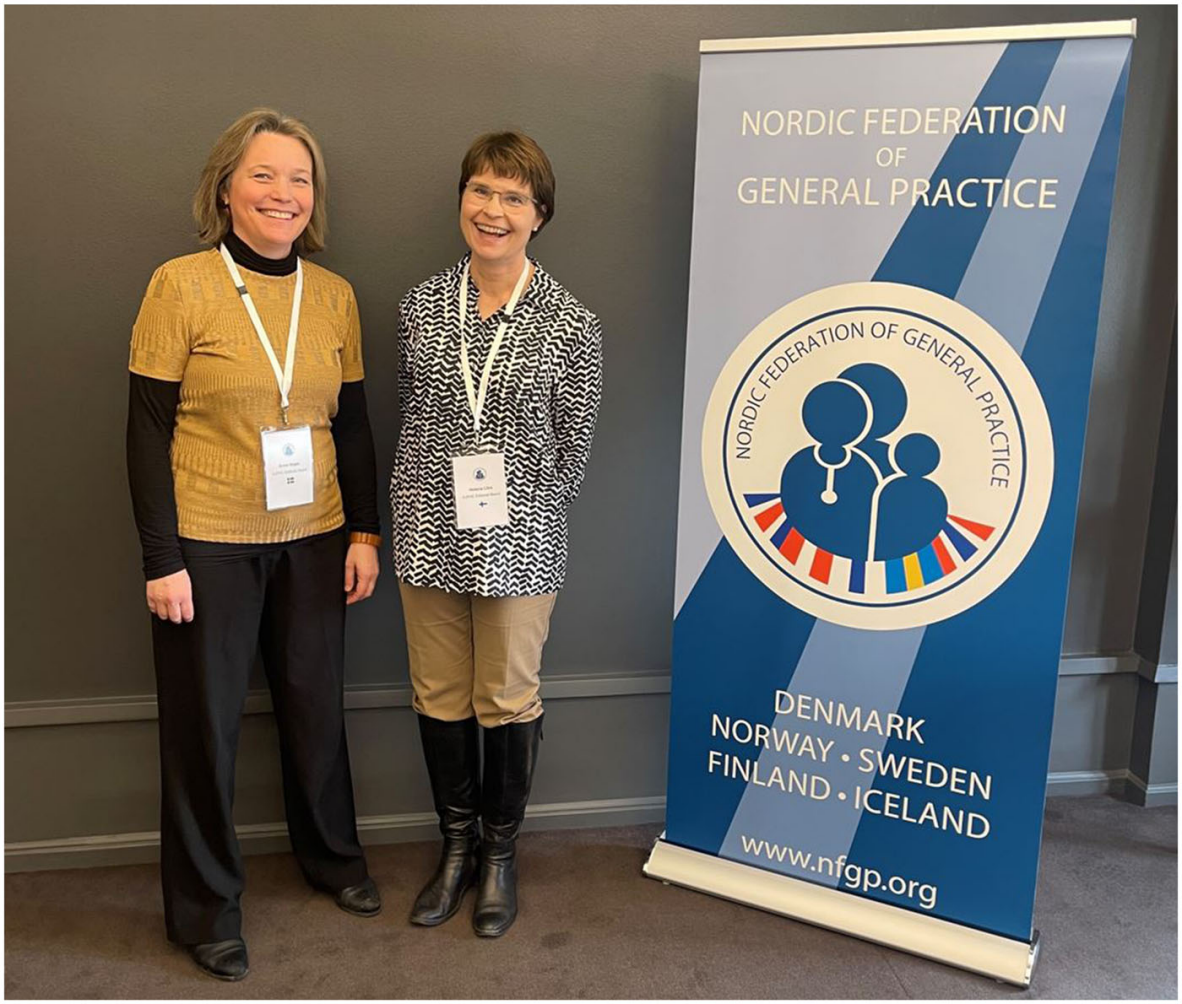# The 40^th^ Anniversary of the *Scandinavian Journal of Primary Health Care*

**DOI:** 10.1080/02813432.2023.2208442

**Published:** 2023-05-11

**Authors:** Johann A. Sigurdsson

**Affiliations:** Nordic Federation of General Practice, Office: Copenhagen, Denmark, and General Practice Research Unit, Norwegian University of Science and Technology (NTNU), Trondheim, Norway

While the history of modern, academic General Practice/Family Medicine (GP/FM) may seem short, the development of our discipline has already reached major milestones. To name just a few: our Danish College celebrated its 50th anniversary in 2020; the World Organization of Family Doctors (WONCA) turned 50 in 2022, and this year, 2023, our Swedish College also celebrates its 50th anniversary [1]. Our discipline is now firmly rooted – scientifically, in terms of our professionalism, and as regards our clear moral intent, vision, and mission [2]. This year we celebrate another landmark – the 40^th^ anniversary of our *Scandinavian Journal of Primary Health Care*. Let’s use this unique opportunity to reflect on our history and look toward our future.

## Why our own journal?

What lay behind the decision our Primary Health Care pioneers made more than 40 years ago to launch a Nordic, English language, medical journal of their own? Various explanations appeared in the journal’s first issue [3]. Here are others which aren’t mentioned there or elsewhere:

In 1966, the doctoral degree thesis that Bent Guttorm Bentsen submitted at the University of Oslo, Norway [4], was rejected by the committee assigned to evaluate it. Bentsen was then a General Practitioner in Nes, an inland Norwegian municipality with only 5807 inhabitants. During the years 1952 to 1956, he had made a thorough, comprehensive study of all those contacting his practice, analyzing the inhabitants’ symptoms, diseases, referral rates, hospitalizations, etc. [5]. He had utilized retrospective methods, classifying medical problems according to the 7^th^ edition of the *International Classification of Diseases* (ICD-7), which then lacked classifications of most symptoms.

This rejection exposed underlying problems. There were too few General Practitioners with academic backgrounds qualifying them to serve on an evaluation committee. Consequently, those who evaluated this GP/FM research study had been enlisted from other specialties and were thus unfamiliar with the relatively new GP/FM discipline.

Furthermore, research into symptoms had not yet been universally accepted, nor had a set of international classifications regarding Primary Care been developed. Little wonder that one of the first actions WONCA took in 1972 was to establish the WONCA *International Classification Committee* (WICC). That very same Bent Guttorm Bentsen, as well as Niels Bentzen (Denmark), were among this committee’s pioneering members. By 1975, the first *International Classification of Health Problems in Primary Care* (ICPC) was published, which soon became a fundamental primary healthcare research tool [6].

During the 1970s, as Nordic countries began shaping the GP/FM academic departments and institutions as well as initiating educational reforms. The collaboration of Nordic General Practitioners became a solid platform.

The impressive range Eof research activities that were already in progress among our Nordic colleagues became clear during our first two *Nordic Congresses of General Practice* – in 1979 in Copenhagen, and in 198E1 in Bergen. At that juncture, our leaders became convinced that a journal of our own was warranted and would be viable. The very next year, 1982, *The Scandinavian Journal of Primary Health Care* was founded [7]. The first issue was published in May of 1983 [3].

## Milestones of the past 40 years

Our journal has been in constant development during the past decades. Here are just two of the many examples of our evolution:

Initially, the ownership and organizational framework of the journal was loosely structured. In 1996, however, a crisis arose regarding collective journal subscriptions. This helped to prompt the 1999 establishment of a formal, Nordic corporate journal structure. Then, in 2005, ownership of the journal was transferred to the newly established *Nordic Federation of General Practice* [7,8].

The second example is the even more consequential shift that began in 2007: our transformation from a paper-based publication into a web-based, open-access journal. This has enabled us to impact Family Practice worldwide.

## Present status and challenges

The last decade has seen our digital journal become firmly established under the leadership of our Editor-in-Chief, Helena Liira, and her editorial staff. According to the parameters of a frequently utilized quality indicator, the journal´s ‘Impact Factor’ (IF) has achieved the current, impressive “3.1” rating.

Other transitions have begun during the last two years (2022-2023). The new contract we have with our publishers offers greater support for expansion. We are also replacing our submission management system, ‘Manuscript Manager,’ with ‘Scholar One’ software. Such shifts not only present a challengingly steep learning curve but also highlight problems. One of these is a chronic shortage of good reviewers. Medical journals everywhere are facing that issue now, and it threatens the quality of all peer-reviewed publications.

### Vision, Mission, and Values

*The Lancet* is also celebrating an anniversary now – its 200^th^! Their Editor-in-Chief, Richard Horton, pointed out in a recent editorial that, from its outset, *The Lancet* was more than a journal [9]. It was an idea, an instrument both for improving the quality of care and for ‘cutting out’ corruption from within that era’s medical establishment. Ergo the name, ‘Lancet,’ which is a double-edged, sharp, surgical knife.

From its inception, our *Scandinavian Journal of Primary Health Care*, too, has been more than a journal. It functions independently, with its own mission, while also being part of a bigger picture: it is one of the cornerstones of the *Nordic Federation of General Practice*. It shares the *Federation*´s ‘Vision and Mission’ as well its ‘Values, Principles and Moral Intent’ [10–14]. As such, our journal and *The Lancet* share common aims: we are both fighting for sustainable values [13] and gender equity, and against corruption, inequality, and overdiagnosis.

### New Editors

This year also brings changes to our editorial staff. Helena Liira, who followed Peter Vedsted as Editor-in-Chief in 2016, is now stepping down. On behalf of the *Nordic Federation*, we’d like to express our sincere gratitude to Liira. She has done an extraordinary job as Editor-in-Chief. She has stood, steadfast, on the front lines, negotiating with our publishers, and scrupulously evaluating the details of our new contract.

We now welcome our incoming Editor-in-Chief, Anna Nager, and her Editorial Board. As Anna comes from Sweden, the editorial office will be moving from Helsinki to Stockholm.

### Our Future

We look forward to continuing to create a sustainable future, with our journal reinforcing our founding ideas and core values [14]. We remain convinced that the knowledge and science we publish results in improved practice, helping to transform our society for the benefit of our patients – who are its citizens.

**Figure:**